# Research highlights, *The Plant Genome*, Volume 14, Issue 3

**DOI:** 10.1002/tpg2.20180

**Published:** 2021-12-04

## 1 MULTI‐TRAIT GENOMIC PREDICTION MODELS

Prediction of breeding values is central to plant breeding and has been revolutionized by the adoption of genomic prediction models, especially machine‐ and deep‐learning‐based models. Sandhu et al. (https://doi.org10.1002/tpg2.20119) optimized multitrait machine and deep learning models for predicting grain yield and grain protein content in wheat using spectral information. This study compares the performance of four machine‐ and deep‐learning‐based unitrait and multitrait models with traditional GBLUP and Bayesian models. Multitrait models performed 0–28.5% and −0.04–15% superior to unitrait models for predicting grain yield and grain protein content. Random forest and multilayer perceptron were the best performing machine and deep‐learning models to predict both traits.

## 2 APPLICATION OF A POISSON DEEP NEURAL NETWORK MODEL FOR THE PREDICTION OF COUNT DATA IN GENOME‐BASED PREDICTION

Genome selection depends on statistical machine learning to perform predictions. For count data outcomes, there are only a few efficient statistical machine learning models for large datasets or for datasets with fewer observations than independent variables. In this paper, Montesinos‐López et al. (https://doi.org/10.1002/tpg2.20118) applied the univariate version of the Poisson deep neural network (PDNN) proposed earlier for genomic predictions of count data. The advantage of the PDNN model is that it captures complex patterns in the data by implementing many nonlinear transformations in the hidden layers. The results from the PDNN model were compared with deep learning models with continuous outcomes, conventional generalized Poisson regression models, and conventional Bayesian regression methods. The results showed that PDNN model outperformed the Bayesian regression and generalized Poisson regression methods in terms of prediction accuracy, although it was not better than the conventional deep neural network with continuous outcomes.

## 3 PANGENOMICS AND MACHINE LEARNING FOR BETTER PLANTS

 Plant breeding continues to benefit from advances in genomics and new technologies. Recent advances in pangenomics and artificial intelligence provide new approaches to accelerate the production of improved varieties. In their new review, Bayer et al. (https://doi.org/10.1002/tpg2.20112) show how pangenomics and machine learning can take plant breeding to the next level. Linking these different fields will lead to improved breeding programs and new varieties adapted to the changing climate.

## 4 DEEP‐LEARNING POWER AND PERSPECTIVES FOR GENOMIC SELECTION

Deep learning (DL) is part of artificial intelligence systems that have been adopted for genomic selection (GS), as they solve complex prediction tasks. Combining GS and DL models can accelerate the use of GS‐assisted plant breeding. Montesinos‐López et al. (https://doi.org/10.1002/tpg2.20122) report that areas of opportunity for using DL methodology in GS are (a) their ability to perform better when solving a large number of problems, (b) easier problem solving because of the automated way in which they perform feature engineering, (c) they represent all layers simultaneously rather than in many steps, (d) their ability to capture complex nonlinear patterns in the data more efficiently because of the inclusion of many hidden layers, and (e) media hype enhancing some remarkable achievements. However, considerable research is required to be able to not only use the existing DL in conjunction with GS but to adapt and develop DL methods that take the peculiarities of breeding inputs and GS into consideration.

## 5 CHARACTERIZATION OF HETEROSIS AND GENOMIC PREDICTION‐BASED ESTABLISHMENT OF HETEROTIC PATTERNS FOR DEVELOPING BETTER HYBRIDS IN PIGEONPEA

Hybrids in crops provide advantages in terms of yield and resistance and tolerance to various stresses. Saxena et al. (https://doi.org/10.1002/tpg2.20125) used a large collection of inbred and parental lines and first‐generation hybrids in pigeonpea to characterize the molecular basis of heterosis and established the molecular foundation of genome‐wide prediction for hybrid breeding programs. They have also identified the loci contributing to mid‐parent heterosis and predicted the best possible combinations for generating high‐yielding cultivars.

## 6 GAIN FROM GENOMIC SELECTION FOR A SELECTION INDEX IN TWO‐ROW SPRING BARLEY

Genomic selection studies are often focused on individual traits, despite the routine use of selection indices in plant breeding programs. Sweeney et al. (https://doi.org/10.1002/tpg2.20138) suggest that genomic selection can improve selection on index values with different weights. Higher realized gain for overall selection index value and some component traits was observed with genomic selection compared with phenotypic selection.  Genetic variance was reduced for traits with a high index weight. Inbreeding was increased with genomic selection compared with phenotypic selection, but restricted mating of high breeding value individuals limited potential inbreeding.

## 7 RAPID COOKING HEALTHY BEANS

Traditional common bean varieties take a long time to cook, and rapid‐cooking beans biofortified with iron and zinc could provide a major boost to human health and nutrition in East Africa. Saradadevi et al. (https://doi.org/10.1002/tpg2.20156) found high genomic heritability for grain yield, cooking time, iron and zinc content in 358 bean varieties in an African bean panel, and therefore genomic selection could rapidly improve these traits. The offspring of optimal contributions selection were predicted to be higher in grain yield, iron and zinc and be faster cooking than the candidate parents. Future rapid‐cooking common bean varieties will improve the health and well‐being of African women and children, while reducing the negative environmental impact and health risks associated with cooking traditional varieties.

## 8 DEEP NEURAL NETWORKS ESTIMATE RELATEDNESS

Recent interest in deep neural networks for genomic prediction has been met with disappointment, as these powerful networks generally underperform classical methods. Ubbens et al. (https://doi.org/10.1002/tpg2.20147) propose that this poor performance is a result of the network estimating relatedness instead of marker effects. It is shown that a network does not lose performance when it has no access to the alleles themselves.

## 9 GENOMIC SELECTION OUTLOOK IN SUGARCANE

Genomic selection (GS) is an important tool for improving the rate of genetic gain and enhancing selection progression during the breeding process in crops. Islam et al. (https://doi.org/10.1002/tpg2.20148) demonstrated that GS could potentially predict the genomic estimated breeding value for selecting the desired germplasm for sugarcane breeding for disease resistance. GS prediction accuracies for brown and orange rust diseases was as high as 0.43 in a three year, three crop cycle field trial. The prediction ability further improved by including a known major gene for resistance to brown rust as a fixed effect in the GS model. It also substantially reduced the minimum number of markers and training population size required for GS study. Both additive and non‐additive genetic effects could contribute genomic sources underlying brown and orange rust.

## 10 GENOME‐ENABLED PREDICTION FOR SPARSE TESTING IN MULTI‐ENVIRONMENTAL WHEAT TRIALS

Sparse testing in genomic‐enabled prediction in plant breeding can be emulated when lines are observed in some environments (overlap) but not in other (nonoverlap). Crespo‐Herrera et al. (https://doi.org/10.1002/tpg2.20151) studied three general cases of the composition of the sparse testing allocation design for genome‐enabled prediction of wheat breeding lines: (a) completely nonoverlapping wheat lines in environments, (b) completely overlapping wheat lines in all environments, and (c) a proportion of nonoverlapping/overlapping wheat lines allocated in the environments. The study used three different genome‐enabled prediction models. Model M1 includes only main effects of environments and lines; M2 had main effects of environments, lines, and genomic effects; and model M3 incorporated the genomic × environment interaction (GE). The results show that M3 captures a large genetic variability and provides higher prediction accuracy than models M1 and M2 for the same allocation design.

## 11 UNBALANCED TRIALS IMPROVED SELECTION EFFICIENCY

The selection gain achieved in crop breeding is limited by the number of environments of a breeding trial by the heritability, which increases with a higher number of environments. But more environments multiply the amount of work and costs, a scarce resource in preliminary trials. Lell et al. (https://doi.org/10.1002/tpg2.20150) report that biometric models that take candidate relatedness into account allow for sparse environmental sampling. In this approach, more environments are included in the trial, but each candidate is only tested in a subset of them. By masking all but 36% of an 11‐environment hybrid wheat yield trial data set, they compared different ways of sparsely testing candidates. When using relatedness‐enabled biometric models, the resulting performance estimates where closely correlated to those of the full unmasked data set, providing a possibility to increase environmental coverage of preliminary yield trials in an economic fashion.

## 12 MACHINE LEARNING IMPROVES PREDICTION ACCURACY

Traits with a complex unknown genetic architecture are common in breeding programs. However, they pose a challenge for selection due to a combination of complex environmental and pleiotropic effects. One such trait, seedling emergence of wheat from deep planting, presents a unique opportunity to explore the best method to use and implement genomic selection models to predict a complex trait. Merrick and Carter (https://doi.org/10.1002/tpg2.20158) showed that non‐parametric machine learning models increased accuracy over common parametric models when combining years but displayed little increase in accuracy over the parametric models in individual years within a breeding program. Further, their own breeding lines displayed comparable accuracy to a diversity panel composed of various Pacific‐Northwest varieties. This showed that breeders can accurately predict and implement genomic selection for a complex trait using machine learning models within their own breeding programs with increased accuracy as they combine trials and environments.

## 13 GENOMIC PREDICTION OF DIVERSE MAIZE ACCESSIONS

Selecting accessions of interest from genebanks requires extensive and expensive evaluation; however, low‐cost genotyping and genomic prediction have allowed us to overcome this obstacle. Recently, Dzievit et al. (https://doi.org/10.1002/tpg2.20160) conducted genomic prediction with the Maize Association Population as the training set. Through cross‐validation, they reported moderate to high predictive ability for 36 traits, which were confirmed using two empirical populations from the Ames Diversity Panel. Besides the precited trait value, an upper bound for reliability (*U* value) was calculated for each accession in the Ames Diversity Panel to quantify the reliability of the prediction. Considering both values is useful for researchers and breeders either searching for accessions of interest with extreme predicted trait values and high *U* values or wanting to identify accessions with modest trait values and low *U* values to capture diversity not represented by the training set.

## 14 GENETICS FOR ADAPTABILITY IN WHEAT LANDRACES

Understanding genetic basis of adaptability of crops to the target environments is paramount. Hanif et al. (https://doi.org/10.1002/tpg2.20096) analyzed collections of landraces from Iran and Pakistan to understand the genetic mechanisms associated with climatic features like ambient temperature, solar radiations, rainfall and vapor pressure. Some common genes were identified which were associated with adaptability to the local environments. This study provides insight into the genetic diversity with emphasis on the genetic architecture of loci involved in adaptation to local environments, which has implications for wheat genetic improvement using landraces.

## 15 CITRUS EVOLUTION IS CHARACTERIZED BY PERVASIVE ADAPTIVE EVOLUTION

Current evidence suggests that the ancestral citrus originated in the foothills of the Himalayas during the late Miocene global cooling (8 million years ago) and immediately diversified into a rapid radiation. The question of how today's enormous citrus diversity was achieved is therefore a very timely one. González‐Ibeas et al. (https://doi.org/10.1002/tpg2.20104) have performed genomic analyses on wild citrus species showing that the main attributes characterizing citrus fruit, namely, the abundance of pectins, terpenoids, and flavonoids, were the result of large expansions of the gene families intimately linked to these metabolites. These changes were invariably associated with selection pressure, tandem duplications, or triplet retention of the gamma event. Extant citrus species also carry important signatures of pervasive adaptive evolution, a phenomenon that very likely contributed to the vast phenotypic differences observed incitrus.

## 16 DOMESTICATION IN CITRUS AFFECTED MAJOR FRUIT ATTRIBUTES AND TRAITS

In contrast to the appealing, seedless, and tasteful fruit of edible citrus, wild pure citrus mandarins bear inedible, small, distasteful, acidic, and seedy fruit. How were these traits so deeply modified during domestication? Gonzalez‐Ibeas et al. (https://doi.org/10.1002/tpg2.20133), through genomic analyses and population genetic tests, have provided original insights on this question. Domestication removed genes involved in the biogenesis of distasteful compounds, increasing palatability and affected genes associated with the regulation of citric acid and modifying pivotal components of citrus flavor. The data also suggest that pummelo introgressions very likely contributed to the increase in ancestral mandarin size. Other key genes of citrus domestication were located in conserved stretches of pure mandarin regions, such as the putative apomictic reproduction gene that allowed huge dispersion of the desirable edible genotypes of citrus.

## 17 GENETIC RELATIONSHIPS EXIST AMONG COWPEA ACCESSIONS OF DIVERSE ORIGINS

Breeding of superior, nutrient‐dense cowpea varieties can contribute to the reduction of hunger and malnutrition in Africa and Asia. This requires diversity in cowpea germplasm for continuous variety improvement. Sodedji et al. (https://doi.org/10.1002/tpg2.20113) observed significant genetic structuration in a large germplasm collection of 274 accessions, which indicates there is potential for improvement. The subgroups consisted mainly of inbred lines but with low genetic diversity. This suggests deploying research efforts to broaden the genetic base of the crop and to utilize the linkage disequilibrium in the germplasm for mapping quantitative trait loci to increase genetic gain in breeding for quality attributes and important agronomic traits in cowpea.

## 18 FIVE STIFF‐STALK MAIZE GENOMES

Modern maize breeding leverages genetic diversity in heterotic gene pools, one of which is represented by stiff‐stalk germplasm. Bornowski and Michel et al. (https://doi.org/10.1002/tpg2.20114) report on the sequencing, assembly, and annotation of the genomes of five key founder stiff‐stalk lines used in U.S. maize breeding programs. Robust, chromosome‐scale assemblies of these five stiff‐stalk genomes, along with the B73 reference genome, which also is a stiff‐stalk, revealed limited dispensable gene content within the stiff‐stalk germplasm. However, comparison of six stiff‐stalk genomes with a non‐stiff‐stalk and Iodent genome revealed a more extensive dispensable genome that potentially contributes to observed heterosis. Retrospective analyses of haplotypes in these stiff‐stalk founder lines with the original breeding population from the early 20th century revealed substantial untapped genetic diversity that can be exploited in future maize breeding.

## 19 DIVERSITY IN MAIZE KERNEL COMPOSITION

Understanding kernel compositional variation and the genetic control of this variation is critical to germplasm improvement efforts, particularly for food‐grade corn applications. Renk et al. (https://doi.org/10.1002/tpg2.20115) show that genetic and environmental factors account for most of the observed variation in compositional traits. There are trade‐offs that exist between compositional traits that may limit gains that can be made for different compositional traits. While some large‐effect loci were identified in the genome that control variation for compositional data, the vast majority of the genetic variation is controlled by many loci of small‐effect size.

## 20 IDENTIFICATION AND MAPPING OF RESISTANCE GENE

Powdery mildew can cause serious yield loss in wheat. Mapping and cloning resistant QTLs is the most feasible means to reduce the disease. Zhan et al. (https://doi.org/10.1002/tpg2.20120) report the identification and mapping of the new powdery mildew resistance gene *PmCH7087* in the introgression line CH7087. Combined RNA‐seq bulked segregant analysis with KASP genotyping, five transcripts were annotated in the spanning 9.68 Mb regions, in which the transcripts of *TraesCS2B01G302800* were involved in signal transduction. Furthermore, *TraesCS2B01G302800.2* was annotated as the closest homologue of serine/threonine‐protein kinase PBS1, a typical participant in the plant disease immune response, indicating that TraesCS2B01G302800 was the candidate gene of *PmCH7087*.

## 21 COMPLETE GENOME SEQUENCE OF MUNGBEAN FOR FUTURE BREEDING PROGRAM

Mungbean is a grain legume cultivated mostly in developing countries in Asia as an excellent cheap source of protein, carbohydrates, folate, and iron. However, despite its nutritional and agronomic importance, mungbean has been an orphan species lacking genetic and genomic resources compared to other legume species. Here, Ha et al. (https://doi.org/10.1002/tpg2.20121) sequenced the genome of mungbean by using the Pacbio platform, resulting in much improved quality of mungbean genome assembly compared with the previously reported assembly. We identified several genetic factors affecting agronomically important traits, especially, synchronous pod maturity. We provide a valuable resource to help the acceleration of genetic gains for key traits to improve yield in mungbean.

## 22 PHYLOGENETICS OF *SORGHUM*

*Sorghum* is a genus with a diverse wild gene pool and complex phylogenetic relationships. Ananda et al. (https://doi.org/10.1002/tpg2.20123), discovered that wild and domesticated sorghum chloroplast genomes have the same gene content and order. The chloroplast and nuclear phylogenies resulted in similar trees with two distinct clusters dividing the genus into two major groups with *Eusorghum*, *Chaetosorghum*, and *Heterosorghum* in one group and *Parasorghum* and *Stiposorghum* in the other. Furthermore, this study highlights the close relationship of the two monotypic subgenera *Chaetosorghum* and *Heterosorghum*. The study defines the diversity of wild sorghums that will be beneficial in crop improvement.

## 23 OPTIMIZING GENOMIC PREDICTION FOR PLANT BREEDING

In plant breeding large numbers of progeny are generated and the aim is to identify the best‐performing ones as potential new varieties. This selection can be assisted by genomic prediction, which is based on the prediction of the performance of untested lines using genome‐wide molecular markers. Zhu et al. (https://doi.org/10.1002/tpg2.20124) report strategies how to optimize this genomic‐assisted selection process. Based on a large soybean panel, the accuracy of this prediction was found to decrease with decreasing relatedness between the lines used to train the prediction model (the training set) and those to be predicted. Single families used as training set showed substantial variation in their prediction accuracy. Combining several families in composite training sets led to an increase in the prediction accuracy compared with the best single family alone. The results highlight the potential of genomic selection for soybean breeding and illustrate the importance of the targeted design of the training set.

## 24 GENE NETWORKS PATTERN MAIZE ENDOSPERM

Normal maize endosperm contains a single layer of epidermis‐like cells called aleurone that have important functions and properties for grain quality. The *naked endosperm1,2* (*nkd1,2*) and *thick aleurone1* (*thk1*), genes regulate the number of cell layers and the differentiation of aleurone cell characteristics. Wu and Becraft (https://doi.org/10.1002/tpg2.20126) performed weighted gene coexpression network analysis to identify how these genes interact and the cellular processes they regulate to control normal cell patterning during endosperm development. The *nkd1,2* and *thk1* genes interact to restrict aleurone cells to a single layer by controlling cell cycle and cell division processes. The *nkd1,2* genes, but not *thk1*, promote aleurone cell differentiation by modulating signaling activity of the phytohormone auxin.

10.1002/tpg2.20127

## 25 PREDICTIVE BREEDING IN HYBRID CROPS

Predictive breeding has changed the classical plant breeding pipeline due to its ability to improve crops more efficiently. Its implementation requires identifying the most effective process for the crop and its breeding scheme. Fonseca et al. (https://doi.org/10.1002/tpg2.20127) assessed different models to predict grain sorghum hybrids based on general and specific combining abilities of elite inbred lines. Results indicate that genomic models can increase prediction accuracy by accounting for the natural population structure existing in the hybrid breeding programs. Additionally, the inclusion of genotype‐by‐environment interaction effects can further improve the accuracy of models. Hence, the application of genomic‐enabled prediction models should become an additional tool for plant breeders to maximize the rates of genetic gain of hybrid crops.

## 26 STATISTICAL MODELING IMPROVES PREDICTION OF WHEAT QUALITY

Measuring end‐use quality of wheat is laborious and expensive. Genetic analysis may serve to predict grain and flour quality. Aoun et al. (https://doi.org/10.1002/tpg2.20128) used statistical modeling to identify specific environments to exclude in genetic prediction models. Genomic prediction accuracies were high for most traits thereby justifying the use of genomic selection to assist breeding for superior end use quality in soft white wheat. Excluding outlier environments based on genetic correlations between environments was more effective in increasing genomic prediction accuracies compared with that based on environment clustering analysis. For kernel size, kernel weight, milling score, ash, and flour swelling volume, excluding outlier environments increased prediction accuracies by 1–11%. However, for grain and flour protein, flour yield, and cookie diameter, excluding outlier environments did not improve genomic prediction performance.

## 27 UNRAVELLING ADULT BARLEY MILDEW RESISTANCE

Barley powdery mildew readily mutates to overcome conventional resistance genes and similarly chemical controls. Focusing on adult plant resistance using landraces and wild relatives of barley, Ge et al. (https://doi.org/10.1002/tpg2.20129) report three major effects, unique resistance genes with distinct features from common resistance genes. These include basal penetration resistance with slight differences between the genes at the cytological level. They are slow mildewing, offering different but effective levels of resistance, and appear to be broad‐spectrum. The new genes offer breeders opportunities to combine powdery mildew resistance genes with different mechanisms.

## 28 GYMNOSPERM PLASTOMES COMPARATIVE ANALYSIS AND TIME DIVERGENCE

Previous studies on gymnosperm plastomes have shown relatively unclear findings. There remains a lack of detailed systematic phylogenetic analyses due to low levels of taxon sampling or the inability to make comparisons with distant relatives. Hence, Lubna et al. (https://doi.org/10.1002/tpg2.20130) determined that plastome size, structure, and gene order were highly variable in the five gymnosperm groups (ginkgo, pines [Conifers I], cupressophytes [Conifers II], cycads, and gnetophytes). The inverted repeats (IRs) in the five gymnosperm groups were evolved through distinctive evolutionary scenarios. Moreover, the IRs were lost in all conifers but retained in cycads and gnetophytes. The divergence between ginkgo and cycads were estimated ∼284 mya and the crown age of cycads was 251 mya. Thus, these findings will allow readers to understand the factors that have the most significant impact on plastomes structure, size variations, IR regions evolutions and functional repeats sequences to solve issues related to gymnosperm phylogeny.

## 29 MAPPING OF ANTHRACNOSE RESISTANCE LOCI IN LENTIL

Anthracnose, caused by *Colletotrichum lentis*, is a devastating disease of lentil (*Lens culinaris* Medik.) in western Canada. Gela et al. (https://doi.org/10.1002/tpg2.20131) identified quantitative trait loci (QTL) and candidate genes associated with anthracnose race 1 resistance in lentil by combining linkage analysis and genome‐wide association study (GWAS). A major‐effect QTL identified on chromosome 3 using linkage mapping was confirmed via GWAS analysis. Across the genome, GWAS identified 14 single nucleotide polymorphisms (SNP) associated with race 1 resistance. Clusters of candidate nucleotide‐binding leucine‐rich repeat (NB‐LRR) and other defense‐related genes were uncovered within the QTL region. The identified SNP markers can be used for marker‐assisted selection (MAS) to advance molecular breeding approaches for improving anthracnose resistance in lentil.

## 30 TRANSCRIPTOME ANALYSIS REVEALS GENES FOR MALE STERILITY IN PIGEONPEA

Discovery of male sterility systems in pigeonpea has paved the way for efficient hybrid seed production. Male sterility induced by the cytoplasm has been optimized for use in a hybrid pigeonpea program. The genes exhibited differential expression between male sterile and male fertile lines of pigeonpea. Results from Bohra et al. (https://doi.org/10.1002/tpg2.20132) suggest impairments in four major pathways including glucose and lipid metabolism, ATP production, pollen development and pollen tube growth, and reactive oxygen species scavenging. The candidate genes and the associated pathways elucidated in the present study provide the foundation to understand the functional genomics of the reproductive development including the male sterility trait in pigeonpea.

## 31 GENETIC AND PHENOTYPIC CHARACTERIZATION OF RICE GRAIN QUALITY TRAITS TO DEFINE RESEARCH STRATEGIES FOR IMPROVING RICE MILLING, APPEARANCE, AND COOKING QUALITIES IN LATIN AMERICA AND THE CARIBBEAN

Rice *(Oryza sativa* L.) is consumed by more than half of the world's population. Rice consumers have very specific quality preferences that outline different markets across the world. Cruz et al. (https://doi.org/10.1002/tpg2.20134) described how molecular tools such as marker assisted selection and genomic selection can be used to develop better rice cultivars for different markets in Latin America and the Caribbean. Their results found four molecular markers that can effectively be used to identify rice lines with different amylose content, gelatinization temperature, and setback viscosity properties. These attributes are used to define different cooking and eating qualities. Also, genomic selection was found to be an appropriate tool for improving grain milling quality traits. These traits are not only controlled by multiple genes but are also very costly to evaluate. These findings created venues to introduce molecular strategies in their breeding toolbox to accelerate the development of high‐quality cultivars.

## 32 GENETIC RESISTANCE CONTROLS WHEAT STRIPE RUST

Durum wheat production is constrained by fungal diseases including stripe rust. Continuous mining of germplasm for the discovery and deployment of stripe rust resistance genes is needed to counter the impact of this disease. In this study, Aoun et al. (https://doi.org/10.1002/tpg2.20136) evaluated a worldwide collection of 432 durum wheat lines to current stripe rust pathogen races in the United States. A total of 32 lines were found to be resistant to all tested races. We also identified known and novel genomic regions associated with stripe rust resistance in this germplasm. This study reports effective sources of stripe rust resistance to contemporary races in the United States and shows that this durum wheat collection is abundant in novel resistance loci that can be transferred into adapted durum wheat cultivars.

## 33 *BAHD‐ACYLTRANSFERASES* IN RICE SUSCEPTIBILITY TO SHEATH BLIGHT DISEASE

A lack of understanding of molecular mechanisms of rice–*Rhizoctonia solani* interaction has hindered the development of resistant cultivars. Owing to the diverse role of *BAHD‐Acyltransferases* in plants, Kumar et al. (https://doi.org/10.1002/tpg2.20140) identified and characterized the members of the rice *BAHD‐AT* gene family using a genome‐wide analysis approach. The RNA‐seq based expression profiling in response to *R. solani* suggested a negative role of *OsBAHD‐ATs* in resistance. Studies on the dynamics of DNA cytosine methylation at *OsBAHD‐AT* loci indicated their epigenetic regulation in response to *R. solani*. Detailed studies on *OsBAHD‐ATs* can help in devising strategies to develop sheath blight‐resistant rice cultivars.

## 34 UNDERSTANDING GENETIC DIVERSITY OF GEORGIA PEANUTS

The cultivated peanut (*Arachis hypogaea* L.) has experienced severe genetic bottlenecks over the course of its evolution and domestication, limiting potential genetic gain. Brown et al. (https://doi.org/10.1002/tpg2.20141) evaluated the genetic diversity of 32 peanut cultivars developed by the University of Georgia breeding program since its inception in 1931. Genotyping‐by‐sequencing revealed a total of 27,142 single nucleotide polymorphisms among these cultivars and a high level of detail for understanding their genetic relationships. The results reported here indicate that part of the success of modern peanut cultivars has likely been due to incorporation of diverse germplasm sources. Quantifying genetic similarity among these cultivars provides a valuable understanding of genetic variation among elite Georgia peanut cultivars that have had a significant impact on the peanut industry within the United States and will serve as a valuable resource for maintaining genetic gain in the 21st century.

## 35 YELLOW RUST RESISTANCE IN WHEAT

Draz et al. (https://doi.org/10.1002/tpg2.20142) used genetic resources of wheat as a source for resistance to yellow rust, a devastating fungal disease in wheat. They evaluated two mapping populations of wheat in the field in Germany and Egypt. Genetic mapping of quantitative trait loci (QTL) yielded a major QTL on chromosome 1B for Population 1 and another QTL on chromosome 6B for Population 2. The wheat reference sequence was used to identify potential candidate genes for the discovered resistance QTL.

## 36 CALLING PLANT REPEATS BY COUNTING DNA WORDS

Repetitive sequences must be identified when analyzing plant genomes. Contreras‐Moreira et al (https://doi.org/10.1002/tpg2.20143) benchmark a two‐step approach that first counts the occurrence of DNA words and then classifies repeats by a comparison with curated sequence libraries. The obtained repeated fractions for 20 angiosperm genomes match those reported in the literature and do not preferentially overlap disease resistance genes. Repeats can be efficiently classified by sequence similarity, provided that an adequate library is available, with the complete protocol taking less than 2 h on a desktop Linux box. A guide to curating your own repeat libraries and the scripts to mask and annotate plant genomes are provided.

## 37 GENETIC CONTROL OF LENTIL PHENOLOGY

Understanding the genetic control of lentil phenology is crucial because its modification is important for regional adaptation. Haile et al. (https://doi.org/10.1002/tpg2.20144) report that *FLOWERING LOCUS T* (*FT*) genes are candidates for controlling flowering in lentil grown in northern temperate environments. A major QTL controlling postflowering development was identified with several senescence‐associated genes within the interval. The flowering‐time QTL was validated in a different genetic background indicating the potential use of the identified markers for marker‐assisted breeding to transfer desirable alleles from unadapted germplasm into elite lentil cultivars without disrupting adaptation.

## 38 QTL FOR SEED SHATTERING AND THRESHABILITY IN INTERMEDIATE WHEATGRASS ALIGN CLOSELY WITH WELL‐STUDIED ORTHOLOGS FROM WHEAT, BARLEY, AND RICE

Perennial grain crops have the potential to improve the sustainability of agriculture; however, few existing species produce enough grain yield to be economically viable. Plant breeders are selecting intensely on yield and domestication traits in the forage species intermediate wheatgrass to develop it as the world's first perennial grain crop for human consumption. Understanding the genetic control of these important traits may expedite the process. Genetic research by Altendorf et al. (https://doi.org/10.1002/tpg2.20145) into three domestication traits, including floret and brittle rachis shattering (loss of seeds at maturity prior to harvest) and threshing ability, revealed that the genetic control of these traits is complex and the results were dependent on families, environments, and analysis method. Regardless, many known genes that confer the same traits in other well‐studied annual grass species, such as barley, wheat, and rice, were implicated as important in intermediate wheatgrass. These results will be used to inform more efficient breeding methodologies.

## 39 GENOMIC REGIONS UNDERLYING SOYBEAN MATURITY GROUPS

Soybean [*Glycine max *(L.) Merr.] is a photosensitive short‐day plant. Soybean maturity has a major impact on yield and other agronomic traits. There were 13 soybean maturity groups (000–IX) defined generally based on the latitude. Using genome‐wide association analyses of soybean genotypes with paired maturity groups and across maturity groups 000–IX, Zimmer et al. (https://doi.org/10.1002/tpg2.20146) identified quantitative trait loci that were unique to specific maturity groups or had an effect across maturity groups. Genome‐wide association analyses across maturity groups 000–IX detected a total of 103 quantitative trait loci and confirmed 54 quantitative trait loci identified in the individual genome‐wide association analysis. Of significant loci identified, nine loci had effects on five to nine maturity groups. Understanding the genetic control of soybean maturity groups will aid breeders to manipulate the maturity loci for genetic improvement of soybean yield.

## 40 POOLED DNA SAMPLES CAN IMPROVE GENOMIC SELECTION

Genomic selection can accelerate yield progress in many breeding programs. Tilhou and Casler (https://doi.org/10.1002/tpg2.20149) reported that pooling and subsetting DNA samples in a switchgrass genomic selection scheme can reduce sequencing effort and improve breeding progress. Models trained using as few as two pooled samples maintained > 80% predictive ability relative to models trained with individual‐scale sequencing.  In addition, genomic estimated breeding values were generated from pooled DNA samples using novel group testing strategies. These strategies can allow a breeder to obtain genomic predictions from greater than one genotype per sequenced DNA sample. Overall, sample pooling and subsetting were able to improve breeding progress relative to individual sequencing, particularly when sequencing budgets were limited.

## 41 GENOMIC SELECTION BEYOND A BREEDER'S OWN PROGRAM

Genomic selection has become a valuable tool for selecting cultivar candidates in many plant breeding programs. Michel et al. (https://doi.org/10.1002/tpg2.20153) show that genomic predictions can also aid in selecting parents and crossing combinations with germplasm developed outside a given breeding program. Augmenting in‐house training populations with externally developed germplasm as well as the usage of multivariate models with secondary correlated and easy to phenotype traits enhanced the accuracy of genomic parent and cross predictions. Genotyping germplasm developed beyond a given breeding program is a convenient way to clarify its relationships with a breeder's own germplasm because pedigree information is oftentimes not available for this purpose. Genomic predictions can thus support a more informed diversity management, especially when integrating simply to phenotype correlated traits to partly circumvent resource reallocations for a costly phenotyping of germplasm from other programs.

## 42 GRASS PEA OLIGOGENIC RESISTANCE TO FUSARIUM WILT

Grass pea plants may be infected by *Fusarium oxysporum* f. sp. *pisi*, the causal agent of fusarium wilt in peas with extensive worldwide yield losses. Although a wide range of responses to this pathogen have been previously reported in grass pea, the genetic basis controlling that diversity of responses was unknown, limiting its breeding exploitation. Through the first grass pea disease resistance genome‐wide association study, Sampaio et al. (https://doi.org/10.1002/tpg2.20154) identified 17 associated SNP alleles, located on pea chromosomes 1, 6 and 7, revealing a grass pea oligogenic control to fusarium wilt. Candidate genes underlying these regions revealed functions related with secondary and amino acid metabolism, RNA, transport, and development. The favorable SNP alleles and putative candidate genes identified in the present study represent important tools to assist precision breeding for grass pea resistance against fusarium wilt.

## 43 GENETIC DISSECTION OF SEASONAL VEGETATION INDEX

Inefficient phenotyping under field conditions has become the bottleneck for crop improvement. Wang et al. (https://doi.org/10.1002/tpg2.20155) demonstrated that unmanned aerial vehicle based high‐throughput phenotyping platforms have great potential for genetic dissection of normalized difference vegetation index (NDVI). Cluster analysis of NDVI obtained at five time points across the growing season classified 1,752 diverse maize accessions into two groups with distinct NDVI developmental trends. Penalized‐splines model was used to obtain genotype‐specific curve parameters and capture the dynamics underlying static NDVI observations. Genome‐wide association study using static NDVI values and curve parameters as phenotypic traits detected signals significantly associated with the traits. Further genetic dissection of projected NDVI values from the penalized‐splines model revealed the dynamic change of genetic effects, indicating the role of gene‐environmental interplay in controlling NDVI across the growing season.

## 44 DRONE AND STATISTICS ACCELERATE BREEDING

In the face of global climate change, technologies are being developed to accelerate crop breeding using knowledge from statistics and data science. In particular, the integration and analysis of DNA data and crop phenotype data is expected to enable more efficient selective breeding than in the past. Toda et al. (https://doi.org/10.1002/tpg2.20157) first used a growth model to efficiently compress soybean growth data obtained by drones. By integrating the compressed information with the DNA data, we were able to predict the phenotype of the crop more accurately than before. By using the predicted values to select plants, fast and effective selection of useful plants will be enabled. Our achievement will help plant breeding to create new cultivars faster than before.

## 45 BULK‐SEGREGANT ANALYSIS FOR WINTER SURVIVORSHIP IN SWITCHGRASS

High winter mortality limits biomass yield of lowland switchgrass planted in the northern latitudes of North America. Twenty‐nine lowland switchgrass populations were evaluated for winter survival and 21 populations with differentialwinter survivorship were used for bulk segregant analysis. Poudel et al. (https://doi.org/10.1002/tpg2.20159) revealed 16 quantitative trait loci (QTL). Many of them were population‐specific, but some were identified in multiple populations. Four QTL (at positions 88 Mb on chromosome 2N, 115 Mb on chromosome 5K, and 1 and 100 Mb on chromosome 9N) that were identified in multiple populations are potentially the most useful QTL. These QTL can be used to accelerate breeding cycles of lowland switchgrass populations.

